# A Neuroanatomical Framework for Upper Limb Synergies after Stroke

**DOI:** 10.3389/fnhum.2015.00082

**Published:** 2015-02-16

**Authors:** Angus J. C. McMorland, Keith D. Runnalls, Winston D. Byblow

**Affiliations:** ^1^Movement Neuroscience Laboratory, Department of Sport and Exercise Science, Centre for Brain Research, The University of Auckland, Auckland, New Zealand

**Keywords:** muscle synergy, stroke, corticospinal tract, proximal–distal patterning, upper limb

## Abstract

Muscle synergies describe common patterns of co- or reciprocal activation that occur during movement. After stroke, these synergies change, often in stereotypical ways. The mechanism underlying this change reflects damage to key motor pathways as a result of the stroke lesion, and the subsequent reorganization along the neuroaxis, which may be further detrimental or restorative to motor function. The time course of abnormal synergy formation seems to lag spontaneous recovery that occurs in the initial weeks after stroke. In healthy individuals, motor cortical activity, descending via the corticospinal tract (CST) is the predominant driver of voluntary behavior. When the CST is damaged after stroke, other descending pathways may be up-regulated to compensate. The contribution of these pathways may emerge as new synergies take shape at the chronic stage after stroke, as a result of plasticity along the neuroaxis. The location of the stroke lesion and properties of the secondary descending pathways and their regulation are then critical for shaping the synergies in the remaining motor behavior. A consideration of the integrity of remaining descending motor pathways may aid in the design of new rehabilitation therapies.

## Definitions

“Muscle synergy” can mean subtly different things, creating the opportunity for confusion. As a biological phenomenon, a commonly accepted general definition of muscle synergy is simply a stable spatiotemporal pattern of activity across muscles simultaneously involved in the performance of a movement. Descending neural activity may result in a net excitation or inhibition of the alpha motor neurons innervating each muscle. If motor neurons of two muscles are excited simultaneously, the muscles are coactivated. Conversely, activity in one muscle may coincide with quiescence in another due to reciprocal inhibition. Natural motor behaviors may result from the additive effect of several synergies. In recent experiments, the term “muscle synergy” has been used to label estimates of synergies derived from quantitative matrix factorization methods applied to simultaneous electromyographic (EMG) measurements (Tresch et al., 2006). The details of the mathematical operation determine specific properties of the synergy estimates extracted. For example, non-negative matrix factorization (NNMF) does not capture inhibitory relationships, which may be a limitation of the method. Another usage of synergy arises in clinical settings, where the term “abnormal muscle synergies” may refer only to the pathological patterns of muscle co-activation that emerge after disruption of the motor system, such as stroke (Brunnström, 1970). This phrasing stems from the fact that pathological synergies are “lower dimensional” than in healthy individuals, hence there are more co-dependencies (synergies) present. In the present article, we adopt the general definition of the term synergy given above, although reference will also be made to clinically abnormal synergies as well as synergies identified by matrix factorization, and the caveats with regard to the definitions of each should be borne in mind.

## Mechanisms of Synergy Formation

To make sense of the ways in which stroke can alter muscle synergies, we need first to appreciate the relationship between the anatomical and physiological basis for synergy formation, and the deficit caused by the stroke, remembering that both acute and chronic changes occur. Abstractly, synergies represent low-dimensional movement information expressed in a higher dimensional space of possible activations. Some synergies may arise purely from functional coordination of high-dimensional structures (“functional synergies”). These functional synergies could be considered “soft” in the sense there are not dedicated anatomical structures existing to subserve them. For example, the spatiotemporal dynamics of upper limb movement change markedly in the context of bimanual tasks, even though the anatomical substrate (for a single side) is identical between unimanual and bimanual conditions (Kelso et al., 1979). Alternatively, synergies may be constructed in synergy-specific anatomical structures and then at some subsequent point in the motor pathway that information would have to diverge to the different muscles. These “anatomical synergies” would be “hard,” in the sense that the combinations of muscles involved will be relatively fixed. Soft synergies resulting purely from functional co-activation are therefore potentially more dynamic and context-dependent than hard synergies.

In healthy humans, the corticospinal tract (CST) is the principal conveyor of voluntary drive to the upper limb (Lemon, 2008). Consequently, it is along this neural pathway that the source of synergies has been proposed. The least flexible hard synergies are presumably expressed by dedicated interneuron networks within the spinal cord. Microstimulation in the spinal cord of frogs [reviewed in Bizzi et al. (2008) or rats (Tresch and Bizzi, 1999)] activates combinations of muscles that depend on the precise stimulation location, generate directed movements, and can be combined to form natural behaviors like jumping and swimming. This result has been taken as evidence of the existence in the spinal cord of anatomical modules that construct hard muscle synergies. Overduin et al. (2012) found that microstimulation of the motor cortex activated combinations of very similar synergies to those observed in natural grasping. That cortical activation gives rise to multiple different synergies suggests that their site of generation lies downstream of the cortex, either in the brainstem or spinal cord.

Mapping studies have been used to identify regions of cerebral cortex connected to a particular muscle, either by direct anatomical tract tracing (Rathelot and Strick, 2006), single cell recording (Schieber and Hibbard, 1993), or assessing functional connectivity with transcranial magnetic stimulation (TMS; Devanne et al., 2006). Instead of the neat, somatotopic arrangement of muscles implied by the motor homunculus concept [which was actually an oversimplification of the reports of Penfield; see Penfield (1954)], maps derived using these methods show that muscle representations on the cortical surface have distributed, complex shapes that overlap with areas connected to other muscles. Overlapping maps are consistent with an anatomical basis for cortical control of hard synergies, since such an architecture means that activation at a single locus on the cortex results in activation of all of the muscles represented at that point, and as the region of activation increases in area, neighboring regions can be recruited in a systematic manner (Wickens et al., 1994; Rathelot and Strick, 2006; Capaday et al., 2013). Distributed muscle representations in primary motor cortex, along with extensive horizontal projections (Huntley and Jones, 1991) may provide a flexible network substrate for soft synergies. A cortical basis for synergies is further supported by the observation that discharge of single corticomotor neurons strongly correlates with activity in a functional set of muscles (Holdefer and Miller, 2002). These different mechanisms and sites of synergy formation, functional, spinal, and cortical, are not mutually exclusive, and it seems likely that all could have effects depending on the context.

**Figure 1:**
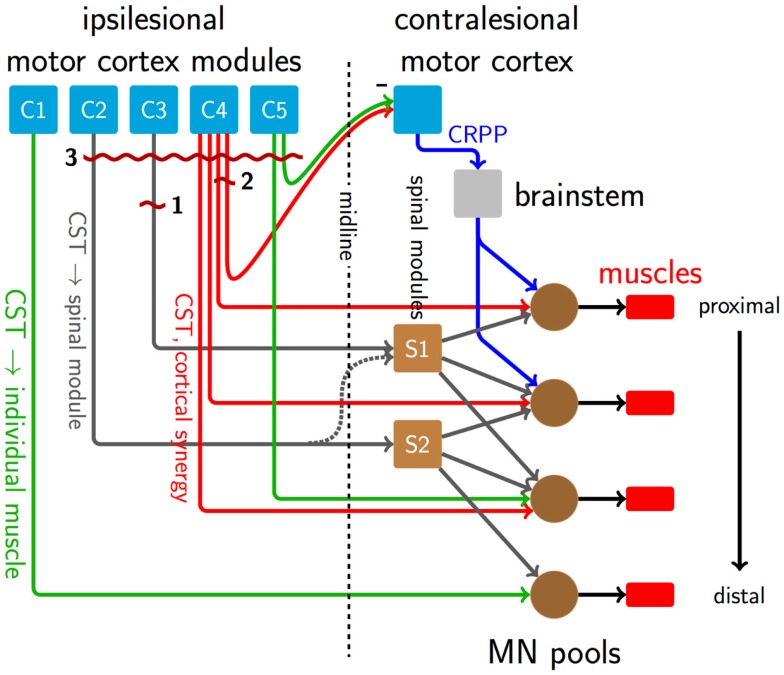
**A schematic of descending pathways involved in the formation of synergies in healthy motor behavior, and their disruption after stroke**. Direct CST connections exist from the cortex to motor neuron pools predominantly for distal muscles, green pathways from C1 to C5. Other CST connections, dark gray pathways from C2 and C3, carry low-dimensional motor commands and innervate synergy-forming spinal modules S1 and S2, which in turn have divergent connections to multiple motor neuron pools at several rostrocaudal levels. Red lines from C4 represent a cortically derived synergy controlling the three proximal motor neuron pools. Connections from C4 and C5 normally inhibit the contralateral motor cortex. Among other functions, this transcallosal input regulates alternate descending pathways like the CRPP (blue lines), which innervates propriospinal neurons linking multiple proximal limb segments. Dark red and wavy horizontal lines indicate sites of damage caused by three stroke lesions. Lesion 1 disrupts low-dimensional information from C3 to the synergy module at S1, and therefore would interfere with activation of the whole synergy mediated by S1, while leaving the synergy structure intact. Lesion 2 represents a mild event, transecting part of the cortically derived synergy from C4, which would alter its structure. Lesion 2 also damages transcallosal connections to the contralesional hemisphere, reducing interhemispheric inhibition and up-regulating the CRPP and other alternate, ipsilateral descending pathways. Since the lesion only affects a small number of CST fibers, remaining descending connections will subsume the damaged functions, and prognosis is good. The up-regulated CRPP interferes with productive CST drive. Suppression of the contralesional hemisphere by non-invasive brain stimulation, for example, c-tDCS, may reduce CRPP activity and thus be beneficial. Lesion 3 is larger than lesion 2, resulting in a severe impairment. Here, the CRPP represents the majority of remaining drive, meaning that c-tDCS of the contralesional hemisphere could be disadvantageous [c.f., Bradnam et al. (2012)].

Figure 1 shows a schematic of motor control structures and descending pathways from the cortex to muscles. C1–5 represent functionally differentiated cortical modules, capturing the repertoire of theorized modes of descending output. These need not correspond to specific anatomical structures, while their relative spatial arrangement is suggestive of the distributed arrangement seen in the cortex, where adjoining regions can represent non-contiguous muscles. C1 and C5 are connected via direct CST fibers to motor neuron pools in the spinal cord. Such individuated cortical connectivity is typical of distal muscles. C4 is similarly connected, but represents a cortical synergy, potentially distinct anatomical regions that are modulated as a unit by common inputs and producing correlated outputs. C2 and C3 connect in a one-to-one fashion to spinal synergy modules (S1 and S2) that each have branching, overlapping connectivity to motor neuron pools. A lateral connection between the descending pathways from C2 to the S1 module is latent (dashed) in the healthy condition. Finally, interhemispheric pathways exist from C4 and C5 to the contralateral motor cortex. The contralateral cortex contains, among others, connections to the brainstem and alternative descending pathways such as the cortico-reticulo-propriospinal pathway (CRPP), which divergently innervate multiple, primarily proximal motor neuron pools.

## Synergies in Healthy Motor Control and after Stroke

The usefulness of the muscle synergy concept is determined by its ability to faithfully describe patterns of muscle activation using fewer dimensions than the number of recorded muscles. In healthy individuals, quantitative methods such as NNMF can identify muscle synergies that account for 75–90% of the variance in the EMG, dependent on the number of muscles recorded from, the complexity of the task performed, and the quantitative methods used to extract them (Sabatini, 2002; d’Avella et al., 2006; Steele et al., 2013). Similar results can be achieved for the hand in isolation (Weiss and Flanders, 2004) despite it having more than 20 kinematic degrees-of-freedom, and approximately 30 muscles.

The most common impairment after stroke is hemiparesis (Wolfe, 2000). Upper limb impairment occurs in 75% of patients, and upper limb paresis is a key indicator as to whether or not patients will engage in activities of daily living 6 months after stroke (Veerbeek et al., 2011). Clearly, this reminds us that recovery of upper limb function is an important goal during rehabilitation (Stinear, 2010). Interestingly, however, a substantial proportion of initial upper limb impairment resolves spontaneously within 3 months post-stroke (Zarahn et al., 2011). Using the Fugl-Meyer (FM) assessment of upper limb impairment, a patient’s change between initial impairment, to impairment at 3 months post-stroke, has been shown to be 0.7× initial impairment, i.e., a fixed proportion. This is known as the proportional recovery rule (Prabhakaran et al., 2008). By 3 months, recovery tends to plateau (Kwakkel et al., 2004) indicative of a spontaneous recovery mechanism. At the time when spontaneous recovery is occurring, the modular organization of muscle synergies remains largely intact (Tropea et al., 2013). The proportional recovery rule is upheld for patients with mild or moderate initial impairment, but not for patients with more severe impairment initially, many of whom recover to a lesser extent than predicted by the rule. Although the proportional recovery rule cannot be used to predict recovery for all patients, it reminds us that most patients are left with some lingering upper limb impairment.

Historically, two main synergies of the upper limb have been identified after stroke. These are the flexor synergy, in which shoulder, elbow, and wrist flexion are obligatorily linked, and the opposite extensor synergy (Twitchell, 1951; Brunnström, 1970). Herein, these are referred to as “abnormal synergies.” The functional consequence of any abnormal synergy is a compromised ability to independently perform daily living activities. Patients with worse initial impairment tend to be those that have the most unresolved impairment by 3 months, and are those who are most likely to express abnormal synergies. Therefore, the emergence of abnormal synergies after stroke may predominantly occur as patients reach the chronic stage, i.e., six or more months after stroke. For this reason, most studies examining abnormal synergies after stroke are conducted at the chronic stage. Much of this disability stems from a loss of independent joint control, which impairs movement and normal access to the workspace. Disturbances in joint control are evident in analyses of both kinematics (Levin, 1996; Reisman and Scholz, 2003) and dynamics (Dewald et al., 1995; Beer et al., 2000). Abnormal synergies likely reflect an emergent property of neural reorganization at multiple levels of the neuraxis, each contributing to the overall composite time course of recovery. Understanding the complex etiology and pathophysiology of abnormal synergies thus requires consideration of both spatial and temporal parameters. Several studies have used NNMF to quantify post-stroke synergies from EMG measurements. Cheung et al. (2009) reported that the underlying structure of muscle synergies is preserved after stroke when patients performed upper limb reaching tasks. In a subsequent study, Cheung et al. (2012) found that the synergies seen in the affected arm of more severely impaired patients could be derived by merging and fractionation of synergies in the unaffected arm, phenomena which could be attributed to an altered activation of synergies by descending commands. More recently, Roh et al. (2013) found that the same number of synergies could explain the data in both healthy control and stroke-affected participants performing an isometric upper limb force production task. However, the structure of synergies involving shoulder muscles were altered with strong co-activation patterns across the anterior medial and posterior deltoid. Several factors may have led to the different conclusions drawn from these studies. Roh et al. (2013) sampled a very chronic group of patients (mean chronicity 15.8 years) performing an isometric task, whereas Cheung et al. (2012) sampled a less chronic but more heterogeneous group (mean 3.0 years) performing gross movement tasks. An informative approach undertaken by García-Cossio et al. (2014) reported differences in the pattern of upper limb synergy expression that were dependent upon the affected anatomical structures (i.e., preservation of sensorimotor cortex). Stroke-affected participants without intact sensorimotor cortex expressed similar synergy patterns in both upper limbs during bilateral gross movement tasks. In patients with a lesion affecting only subcortical structures, the affected limb expressed a greater number of synergies and less similar synergies relative to the ipsilesional limb. The degree of similarity, or preservation, of synergies between limbs was positively related to hand function in the group with intact sensorimotor cortex. These results highlight the important but non-exclusive contribution of motor cortical areas to synergy expression and demonstrate that compensatory synergy formation in the intact motor cortex is potentially maladaptive.

Cortical mapping also provides insight to reorganization after stroke. In animal models of stroke, extensive evidence of cortical reorganization has been shown using intracortical microstimulation, whereby distal areas affected by the experimental lesion emerge in motor cortical areas normally dedicated to proximal representation, presumably as a result of use-dependent plasticity (Nudo et al., 1996). Using TMS mapping, Byrnes and colleagues found that some patients with upper limb deficits at the chronic stage after stroke had shifts of intrinsic hand muscle representations in the mediolateral axis, suggesting reorganization within primary motor cortex (M1). Such effects might be seen after lesion 1 in Figure 1, where a small lesion leaves substantial cortical and descending substrate intact, suitable for reorganization. Other patients had shifts along the antero-posterior axis, indicative of recruitment of cortical neurons in secondary motor areas known to have direct connections to the spinal cord (e.g., lesion 3, Figure 1) (Byrnes et al., 2001). These mutually exclusive patterns of reorganization remind us of the considerable redundancy in the motor system.

The effect of a stroke on healthy muscle synergies will depend on whether the level of the lesion in the motor pathway was up- or downstream of the point of divergence of low- to high-dimensional information. Interference above the point of divergence would alter the ability to recruit whole synergies whereas disruption below the point of divergence would alter the structure of individual synergies themselves. Hemiplegia is predominantly associated with subcortical lesions of the posterior limb of the internal capsule (PLIC), directly damaging the descending CST. PLIC lesions would be downstream of the point of divergence of cortically derived synergies (as in the case of lesion 2 in Figure 1), and therefore interfere with the composition or weightings of the synergies, as seen in Roh et al. (2013). PLIC lesions would also be upstream of synergies constructed in the spinal cord (lesion 1, Figure 1), a point at which they should alter the activation patterns of whole synergies at a time. Merging and fractionation of synergies reported by Cheung et al. (2012) could be explained by altered activation of whole synergies, rather than changes to their internal structure.

## A Neurophysiological Synergy Task

Gerachshenko et al. (2008) presented an alternative way of looking at synergies after stroke by using TMS of M1 and recording motor evoked potentials (MEPs) in the paretic upper limb muscles. They computed a “synergy ratio” (SR), comparing the size of MEPs in biceps brachii (BB) when acting as a task antagonist compared to that when acting as an agonist. MEPs were collected using TMS intensity close to motor threshold, in the 100–200 ms before isometric forearm pronation, when BB acts as an antagonist, and before isometric elbow flexion when BB is an agonist. In healthy individuals, the SR is normally around 0.3 because corticomotor excitability of BB is suppressed prior to pronation, presumably by a central feed-forward mechanism that reflects a reciprocal inhibition synergy between the BB and pronator muscles (Gerachshenko and Stinear, 2007). After stroke, the SR is much larger (with some patients having SR values of 1.0 or above), because suppression of BB excitability before pronation is reduced (Gerachshenko et al., 2008). The larger SR reflects a reduced selectivity in BB activation with SR correlating with upper limb impairment as assessed using the upper limb FM assessment for these patients.

What neural pathways mediate the loss of selective suppression of antagonists that may contribute to abnormal synergies after stroke? Schwerin et al. (2008) found that the presence of ipsilateral MEPs in proximal muscles such as pectoralis major were associated with upper extremity FM scores in patients at the chronic stage after stroke, so up-regulation of uncrossed descending pathways from the contralesional hemisphere is a candidate mechanism. An idea proposed by Bradnam et al. (2013) was that the reduced precontraction suppression in the study by Gerachshenko et al. (2008) was mediated, at least in part, by reduced transcallosal inhibition of the contralesional hemisphere leading to an increase in contralesional M1 (cM1) excitability (Murase et al., 2004). The resulting increase in cM1 excitability up-regulates a CRPP, which has ipsilateral projections to the upper limb via propriospinal neurons. These interneurons are found rostrocaudally in the spinal cord linking motor neuron pools across segments of the cervical spine, and are therefore a potential source of primitive synergistic connections between muscles.

To test this idea, McCambridge et al. (2011) applied transcranial direct current stimulation [tDCS; for review see Liew et al. (2014)] to suppress M1 excitability in the hemisphere ipsilateral to the arm performing the SR task, with the aim of improving selectivity in the ipsilateral BB. They found in healthy subjects, cathodal tDCS (c-tDCS) produced the beneficial effect of decreasing SR compared to sham tDCS. The size of the improvement in SR was negatively correlated with the pre-intervention SR level (McCambridge et al., 2011). These results indicated that c-tDCS might down-regulate descending ipsilateral input to motor neurons innervating the proximal upper limb, and may therefore be beneficial for reducing the expression of abnormal synergies after stroke.

The effect of c-tDCS suppression of the contralesional hemisphere was examined in the context of an SR task, with 12 patients after first-ever subcortical stroke (Bradnam et al., 2012). After stroke, the degree to which the contralesional hemisphere may contribute to control of the paretic upper limb likely varies with stroke severity. That is, mildly impaired patients likely revert to predominantly contralateral control of the paretic upper limb, as is the case in healthy adults. Conversely, patients with more severe impairment are more likely to rely on ipsilateral control from the contralesional hemisphere, as descending pathways from the lesioned hemisphere may be no longer viable. The authors quantified motor pathway integrity using diffusion-weighted MRI and measures of fractional anisotropy at the level of the posterior limbs of the internal capsules. In mild and moderately affected patients, c-tDCS of the contralesional motor cortex tended to improve the SR measured in the paretic upper limb. However, in more severely impaired patients (FM score < 45), SR was worsened after c-tDCS. The results were striking in that the direction and extent of SR change after c-tDCS correlated with impairment, spasticity, and fractional anisotropy measures. This reminds us that the non-stroke hemisphere may contribute to upper limb synergies and impairment after stroke, and, that non-invasive brain stimulation protocols such as c-tDCS are unlikely to be “one-size-fits-all” (Bradnam et al., 2013). Patients who have to rely on these alternative pathways have reduced capacity for recovery and are often left with lingering impairment. It is our contention that this impairment reflects the limited ability of ipsilateral or “alternate” pathways to replace the loss of individuated control afforded by the damaged CST.

## Take-Home Messages and Future Directions

Synergies that arise after stroke are shaped by two complementary mechanisms: the disruption of healthy synergies by the lesion and the development of new synergies by cortical reorganization and the unmasking and up-regulation of alternative descending pathways. No two strokes are the same and both of these processes are determined by the location and size of the lesion. Thus, the potential for recovery of motor function depends on which anatomical structures remain intact and the extent to which neuroplastic processes can remap low-dimensional movement information to the high-dimensional activation space utilizing those remaining anatomical substrates.

At present, gross analysis of structural damage to the PLIC informs the prognosis for recovery of upper limb function (Stinear et al., 2012), and indicates the suitability of non-invasive brain stimulation as a therapeutic adjuvant to physical rehabilitation (Bradnam et al., 2012). Higher resolution imaging of the lesion may facilitate increased diagnostic and prognostic specificity. However, this information will only be useful in the context of a more refined understanding of healthy corticomotor physiology. Improved anatomical knowledge such as the somatotopy of the CST through the PLIC (Holodny et al., 2005) and a more complete knowledge of the structure and function of alternate pathways will be required to better assess the potential for recovery of function (Stinear et al., 2007). More accurate, individualized prognoses will inform more realistic expectations for recovery, which can be used to help therapists, patients, and their families set realistic goals at the time when rehabilitation begins (Stinear et al., 2012).

A better understanding of the neuroanatomical structures in the cortex and spinal cord that underlie synergy formation, as well as the factors that regulate their maintenance and modification may improve rehabilitation practices. For example, the prescription of physical therapy might be guided to develop useful synergies that maximize functional movement capacity while avoiding reinforcement of maladapted movement patterns.

Synergies represent a useful concept for the investigation of upper limb movement affected by stroke. The effect of a stroke lesion on muscle synergies depends critically on its size and location relative to descending motor pathways. This rule affects both direct short-term effects of the lesion and long-term adaptive or maladaptive neuroplastic changes that may involve alternate descending pathways. A more complete understanding of the structures involved and the mechanisms by which they generate synergies may enhance future clinical practice, better enabling rehabilitation professionals to devise treatment plans based on the residual capacity of the descending motor pathways of the individual patient.

## Conflict of Interest Statement

The authors declare that the research was conducted in the absence of any commercial or financial relationships that could be construed as a potential conflict of interest.

## Abbreviations

BB, biceps brachii; cM1, contralesional M1; CRPP, cortico-reticulo-propriospinal pathway; CST, corticospinal tract; c-tDCS, cathodal tDCS; EMG, electromyography; FM, Fugl-Meyer assessment; M1, primary motor cortex; MEP, motor evoked potential; MRI, magnetic resonance imaging; NNMF, non-negative matrix factorization; PLIC, posterior limb of the internal capsule; SR, synergy ratio; tDCS, transcranial direct current stimulation; TMS, transcranial magnetic stimulation.
